# State-Dependent Synchrony and Functional Connectivity in the Primary and Secondary Whisker Somatosensory Cortices

**DOI:** 10.3389/fnsys.2021.713397

**Published:** 2021-09-20

**Authors:** Mohamed Khateb, Jackie Schiller, Yitzhak Schiller

**Affiliations:** ^1^The Rappaport Faculty of Medicine, Technion – Israel Institute of Technology, Haifa, Israel; ^2^Department of Neurology, Rambam Medical Center, Haifa, Israel

**Keywords:** synchrony, functional connectivity, brain states, barrel cortex, secondary somatosensory cortex

## Abstract

Synchronized activity plays an important role in sensory coding and memory and is a hallmark of functional network connectivity. However, the effect of sensory activation on synchronization and cortical functional connectivity is largely unknown. In this study, we investigated the effect of whisker activation on synchronization and functional connectivity of the primary (wS1) and secondary (wS2) whisker somatosensory cortices at the single-cell level. The results showed that during the spontaneous pre-stimulus state, neurons tended to be functionally connected with nearby neurons which shared similar tuning characteristics. Whisker activation using either ramp-and-hold stimulation or artificial whisking against sandpaper has significantly reduced the average overall pairwise synchronization and functional connectivity within the wS1 barrel and wS2 cortices. Whisker stimulation disconnected approximately a third of neuronal pairs that were functionally connected during the unstimulated state. Nearby neurons with congruent tuning properties were more likely to remain functionally connected during whisker activation. The findings of this study indicated that cortical somatosensory networks are organized in non-random small world networks composed of neurons sharing relatively similar tuning properties. Sensory whisker activation intensifies these properties and further subdivides the cortical network into smaller more functionally uniform subnetworks, which possibly serve to increase the computational capacity of the network.

## Introduction

Synchronization of neural activity is a fundamental feature of the cortical network. Neuronal responses in the cortex are correlated on spatio-temporal scales ranging from slow blood oxygenation level-dependent (BOLD) signals to oscillations of the extracellular field potentials. The correlation range goes up the action potential firing and intracellular membrane potential of individual neuronal pairs. Synchrony has been shown to participate in various physiological processes, including coding of sensory information, motor control, perceptional integration, attention signaling, and memory processes (Riehle et al., 1997**;** Dan et al., 1998; Roy and Alloway, 1999; Steinmetz et al., 2000; Engel et al., 2001; Fries et al., 2001; Buzsáki and Draguhn, 2004; Zhang and Alloway, 2004; Poulet and Petersen, 2008; Bruno, 2011; Hansen and Dragoi, 2011; Lobier et al., 2018; Singer, 2018; Petersen, 2019). Synchrony was also widely used to define functional connectivity within the brain neural network using either resting-state BOLD MRI signals (Blatow et al., 2007; Wang et al., 2013; Gordon et al., 2017), EEG and ECoG signals (Hummel and Gerloff, 2005; Rose and Büchel, 2005; Schoffelen and Gross, 2009), and pairwise correlated firing of individual neurons (Temereanca et al., 2008; Bruno, 2011; Constantinople and Bruno, 2011).

Rodents are equipped with an array of vibrissae on their snout, i.e., mystacial vibrissae, which serve as highly efficient tactile somatosensory devices. The tactile information obtained by whiskers is conveyed to the primary somatosensory barrel cortex (wS1), and a lesser degree to the secondary barrel cortex (wS2) *via* the thalamus (for a review, see Diamond and Arabzadeh, 2013; Feldmeyer et al., 2013; Adibi, 2019). Similar to the other brain regions, synchrony at the single-cell correlated action potential level has been reported previously in the wS1 cortex (Roy and Alloway, 1999; Zhang and Alloway, 2004). However, the functional connectivity based on pairwise correlated firing within the wS1 and wS2 cortices has not been systematically studied. Thus, the effect of whisker activation on the functional connectivity of wS1 and wS2 is largely unknown. In this study, we investigated these questions using multi-electrode single-unit recordings from multiple neurons located in different layers of the wS1 and wS2 somatosensory cortices of anesthetized rats. The results showed that whisker activation by either the passive ramp-and-hold stimulation or artificial whisking against sandpaper has decreased pairwise synchronization and disconnected functional connections between neuronal pairs in a distance and tuning-dependent manner. Therefore, whisker activation resulted in a smaller world network that preferred functional connectivity between neurons sharing similar tuning properties in somatosensory cortices.

## Materials and Methods

### Surgical Preparation for Recording in Anesthetized Rats

The experiments were conducted in accordance with the National Institutes of Health (NIH) and institutional standards for the care and use of animals in research and received the approval of our institutional animal ethics committee as described in the previous study (Khateb et al., 2017). The study experimented with P30-35 Wistar rats which were anesthetized by intraperitoneal injection of urethane (20% dissolved in normal saline, a dose of 1.6 g/kg). Prior to surgery, 2% of lidocaine was applied locally over the scalp. In artificial whisking experiments, the bucco-labialis branch of the facial nerve which innervates muscles in the whisker pad was exposed and severed at its proximal segment. The skull was exposed and well cleaned. A craniotomy of 2–3 mm^2^ was drilled over the S1 barrel cortex at 2.5 mm posterior to bregma, 4.5 mm lateral to the midline for S1; the same was done to the S2 cortex at 2.5 mm posterior to bregma, 7.5–8 mm lateral to the midline for S2. A well that surrounded the craniotomy was constructed using dental cement. In addition, a metal plate was glued to the skull rostral to the dental cement well and later used to hold the head of the rats in place. After the dental cement was constructed, the dura mater was carefully removed over a small area at less than 1 mm^2^ to allow access to the cortex. The well which surrounded the craniotomy was filled with artificial cerebrospinal fluid (ACSF) containing 125 mM NaCl, 25 mM NaNCO_3_, 25 mM Glucose, 3 mM KCl, 1.2 mM NaH2PO_4_, 2 mM CaCl_2_, and 1 mM MgCl_2_ saturated with 95% O_2_ and 5% CO_2_ pH 7.4. Body temperature was carefully maintained at 36–37°C using a heating pad (FHC, Montana, United States).

To identify the principal barrel, we initially performed optical intrinsic imaging as described in the previous studies (Garion et al., 2014; Khateb et al., 2017). The location of the principal barrel was later used to guide electrode insertion.

### Electrophysiological Extra-Cellular Single-Unit Recordings

Electrophysiological recordings were performed simultaneously from multiple neurons in layers 2–5 of the wS1 or wS2 cortices using silicone multi-contact probes as described in a previous study (Khateb et al., 2017). The probe (1 × 16 MEA, NeuroNexus, Michigan, United States) which was used in this experiment was composed of a single shaft linear probe containing 16 recording contacts separated by 50 μm. We positioned the probe such that the first contact will be located in layer 2 at 200–300 μm below the pia. Thus, the last contact was positioned in layer-5 950–1,050 μm below the pial surface. Electrodes were inserted into the wS1 or wS2 cortices using a stereotactic micro-manipulator (TSE, Bad Homburg, Germany). After electrode insertion, we verified the identity of the principal whisker (in S1 experiments) or whiskers (in S2 experiments) by multi-unit activity during manual deflection of the principal whisker (wS1) or whisker row (wS2). We trimmed all whiskers aside from the principal whisker for wS1 recordings or whisker row for wS2 recordings. Electrophysiological data were acquired with the 16-channel ME-16 system and MC Rack software (Multichannel Systems, Reutlingen, Germany). The recorded data which was acquired at 25 kHz was initially amplified at ×1,000 and stored in the computer. The analysis was performed primarily offline. In addition, the data was filtered online at 1–5 kHz and displayed to allow for online monitoring of the unit activity during the experiments. For the offline analysis, the raw recorded data was replayed and filtered at 1–5 kHz to obtain the unit activity.

For offline sorting, we initially detected events with peak amplitudes at >3.5 *SD* of the baseline value. These events served for multi-unit analysis (MUA). For single-unit analysis, we further sorted the threshold events through semi-automatic clustering algorithms using the offline spike sorter from Offline Sorter^TM^ (OFS) (Plexon, TX, United States), followed by the manual verification and correction of these clusters, if needed, as described in a previous study (Khateb et al., 2017).

The sorted spike trains were further analyzed using the Neuroexplorer software (Nex Technologies, Alabama, United States) and home written software in MatLab (MathWorks, MA, United States). Similar to the previous studies from the barrel cortex, we usually recorded from 2–3 units per contact after exclusions (Rousche et al., 1999; Lebedev et al., 2000; Panzeri et al., 2001). The average signal-to-noise ratio (SNR) value of our units was 11.2 ± 0.4 with a previously reported range of 7–15 (Rousche et al., 1999).

Clusters were accepted as single units if all the criteria were met. The first criterion was if the waveform shape remained consistent and stable throughout the recording. This was verified by the “Sort-Quality vs. Time” analysis in the OFS software. Units were excluded in case the average amplitude or half-width of the unit changed significantly (ANOVA test) between the initial and last 20% of recorded spikes. The second among the criteria was if the firing rate was >0.5 Hz, as neurons with low firing rates lacked the statistical power to reliably calculate cross-correlograms. The third criterion was if the inter-spike interval (ISI) was >2 ms to avoid the noise and recordings from two different neurons. The fourth among the criteria was if the ISI distribution showed a smooth exponential-like curve. The fifth and most important criterion is a statistical criterion of *p* < 0.05 (multivariate ANOVA) of cluster separation in the 2D planes as well 3D spaces. The sixth and last among the criteria was if the cluster validity was confirmed using four statistical tests, including Dunn 1, Davies Bouldin indexes, pseudo-f, and J3 statistics. In Dunn 1 validity index, values >2 indicated well-sorted channels while >1 indicated intermediate sorting quality. As for the Boulding index, values below 0.3 implied well-sorted channels while below 0.5 implied intermediate sorted units. Pseudo-F was >100,000 for well-sorted channels while >50,000 for intermediate sorting quality. Lastly, a higher J3 ratio indicated better clustering and well-separated units, wherein values above >2 indicated well-sorted channels and >1 indicated intermediate sorting quality. In addition to a significant *p*-value of the multivariant ANOVA of cluster separation, units were accepted if at least two among the above discussed four tests yielded a high degree of sorting while the other two tests demonstrated at least intermediate sorting quality. Units that showed >0.9 cross-correlogram values were excluded to eliminate noise. The sorted spike trains were further analyzed using the Neuroexplorer software and home written software in MatLab.

To quantify synchrony and functional connectivity, we calculated the pairwise cross-correlation function and Pearson’s correlation coefficient for all possible pairs in our recordings. Neuronal pairs with significantly correlated firing were defined as functionally connected. More specifically, we defined neuronal pairs as functionally connected if they fulfilled all of the three following criteria: (1) Pearson’s correlation coefficient values of 0.2 or greater during the spontaneous and evoked states which yields a *p* < 0.05 (Narayanan and Laubach, 2006); (2) peak cross-correlogram value greater than two *SD* above the cross-correlogram mean during the spontaneous and evoked states (Maldonado et al., 2000); (3) overall spike count which was above 3,000 during the recording session (Narayanan and Laubach, 2006), with at least 1,000 spikes during both the spontaneous and evoked states.

All the averaged results were presented in box plots which displayed the median (solid line), mean (asterixis), the two middle quartiles (rectangle), 1.5× inter-quartile value or lower value (lower whisker), 1.5× inter-quartile value or upper value (upper whisker), and outlier values (circles). Statistical testing was performed using the paired and unpaired Student’s *t*-test or ANOVA whenever the data adhering to a normal distribution and parametric statistical tests were feasible. When the data did not adhere to a normal distribution as examined by the Chi-square goodness of fit test, we otherwise used the Mann-Whitney or Kruskal Wallis non-parametric tests. In addition, the Chi-square test was performed for comparing histograms. Statistical tests were performed using Prism (GraphPad Software, San Diego, CA United States^1^) and Excel software (Microsoft Corporation, 2016).

A fluorescent dextran (fluorescent dextran-A solution of 2 mM fluorescent dextran Alexa-488 or Texas Red; Invitrogen, United States) was injected into the electrode tract using a pressure injector at the end of the experiment to verify the recording location. Later, the rat was sacrificed and trans-cardially perfused with 4% paraformaldehyde for histological processing. The location of the fluorescent dye was compared to the barrel field for S1 experiments. For S2 experiments, the location was determined by comparing coronal slices to anatomical features presented in stereotactic rat brain atlas.

### Vibrissa Stimuli

We used two different passive stimulation paradigms applied to the principal vibrissa:

1. Passive ramp-and-hold stimulation of the principal vibrissa. The principal vibrissa was rapidly deflected (1,300°/s) with a single ceramic piezoelectric bimorph for a period of 200 ms as described in the previous studies (Lavzin et al., 2012; Khateb et al., 2017). To avoid ringing of the vibrissa during the rapid deflection phases, we generated a sigmoidal onset and offset of the ramp-and-hold pulses as described in a previous study (Khateb et al., 2017). In most of the experiments, the ramp-and-hold stimulation was performed in one direction. To investigate angular tuning, a passive ramp-and-hold deflection of the principal vibrissa to eight different directions was performed to identify the angular tuning of neurons, as described in the previous studies (Lavzin et al., 2012; Khateb et al., 2017). With this stimulation paradigm, the principal vibrissa was deflected in the eight different directions separated by 45° (0°, 45°, 90°, 135°, 180°, 225°, 270°, and 315°) with 200 ms ramp-and-hold stimuli using two perpendicular pairs of ceramic piezoelectric bimorphs. Stimuli were delivered at 0.5 Hz to prevent steady-state adaptation of vibrissa-evoked responses. We repeated the ramp-and-hold vibrissa deflection to each angle direction 50 times. The repetitions for each direction were divided into two equal blocks and the different deflection blocks were applied in random order.
2. Artificial whisking against sandpaper. This stimulation was performed as described in the previous studies (Garion et al., 2014; Khateb et al., 2017). We exposed and stimulated the bucco-labialis branch of the facial nerve to induce artificial whisking movements. The bucco-labialis nerve was cut and its distal end mounted on a pair of bipolar tungsten electrodes. We applied a train of 10 protraction-retraction cycles applied at 5.5 Hz. For each cycle, a protraction was induced by a train of pulses at 0.5–4 V, 40 μs duration which was applied through an isolated pulse stimulator (A360, WPI) at 100 Hz, followed by a passive vibrissa retraction. The tip of the principal vibrissa (wS1) or the whisker row (wS2) contacted a piece of sandpaper with different coarseness (P120, P320, P600, P1000, and smooth surface) where the vibrissa brushed against during the artificial whisking. Artificial whisking was visually monitored under a stereomicroscope to ensure proper movements of the vibrissa.

## Results

### Synchronization and Functional Connectivity of the wS1 Cortex During Spontaneous and Sensory-Evoked States

**FIGURE 1:**
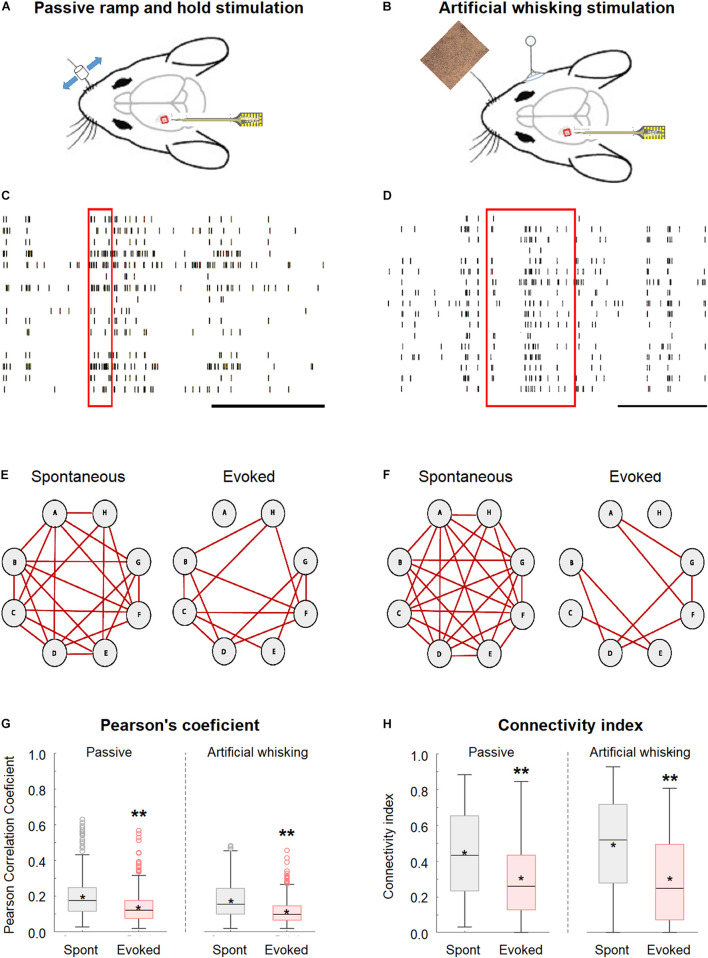
Desynchronization during evoked activity in the primary barrel vWS1 somatosensory cortex. **(A,B)** Scheme of the experimental design with the recording electrode in wS1 and the principal whisker is activated either with ramp-and-hold passive vibrissa deflection **(A)**, or artificial whisking stimulus against sandpaper **(B)**. The mouse was adapted from SciDraw caricatures (doi.org/10.5281/zenodo.3925903). **(C,D)** Examples of the raw spike train recorded during the passive ramp-and-hold whisker deflection **(C)** and artificial whisking against sandpaper **(D)**. The red rectangles represent the time of stimulation, while the black scale bar at the lower right corners represents 1 s. **(E,F)** Individual examples of connectivity diagrams of eight-layer 2-3 neurons during spontaneous pre-stimulation (left) and evoked activity (right) in the passive ramp-and-hold whisker deflection **(E)** and artificial whisking against sandpaper **(F)**. The red lines designated functionally connected neurons. The two examples are from two different rats. **(G)** Box plots of the pairwise Pearson correlation coefficient from vWS1 during spontaneous pre-stimulus (gray) and evoked (red) activities in the passive ramp-and-hold whisker deflection (left, Passive) and artificial whisking against sandpaper (right, Artificial whisking). The middle line in the box plots represents the median value and the asterixis represents the mean value. **(H)** Boxplots of the connectivity index from wS1 neurons during spontaneous pre-stimulus (gray) and evoked (red) states during the passive ramp-and-hold whisker deflection (left, Passive, 213 neurons in 7 rats) and artificial whisking against sandpaper (right, Artificial whisking, 209 neurons in 7 rats). A value of **p < 0.01 was obtained with the *t*-test. *p < 0.05.

This study investigated the effect of whisker activation on neural synchrony and functional connectivity of the wS1 cortex through simultaneously recording the single-unit activity from multiple neurons located in different neocortical layers of wS1 (depth of 200–950 μm from the pia surface) using multi-contact single shaft electrodes (length of 750 μm and inter-contact distance of 50 μm) while stimulating the contralateral whiskers (Figures 1A–D). The whiskers were stimulated by either the passive piezo ramp-and-hold deflections (Figures 1A,C) or artificial whisking against sandpaper (Figures 1B,D). To quantify synchrony and functional connectivity, we calculated the pairwise Pearson’s correlation coefficient for all possible pairs in our recordings. Based on this parameter, we determined which of the pairs were functionally connected. We defined neuronal pairs as functionally connected if they fulfilled all the three following criteria. The first criterion involved the Pearson’s correlation coefficient values of 0.2 or greater which should yield a *p* < 0.05 (Narayanan and Laubach, 2006). The second criterion included the peak cross-correlogram value as greater than the two *SD*s above the cross-correlogram mean (Maldonado et al., 2000). The third criterion involved the overall spike count above 3,000 during the recording session (Narayanan and Laubach, 2006), with at least 1,000 spikes during both the spontaneous and evoked states. Based on this data, we calculated the *connectivity index* for each neuron; this index represented the percent of functionally connected neurons out of the total recorded neurons.

The whisker activation with either the passive ramp-and-hold stimulation or artificial whisking against sandpaper significantly decreased the network synchrony and functional connectivity in the wS1 cortex as compared to the spontaneous pre-stimulus state (600 ms preceding stimulation).

**FIGURE 2:**
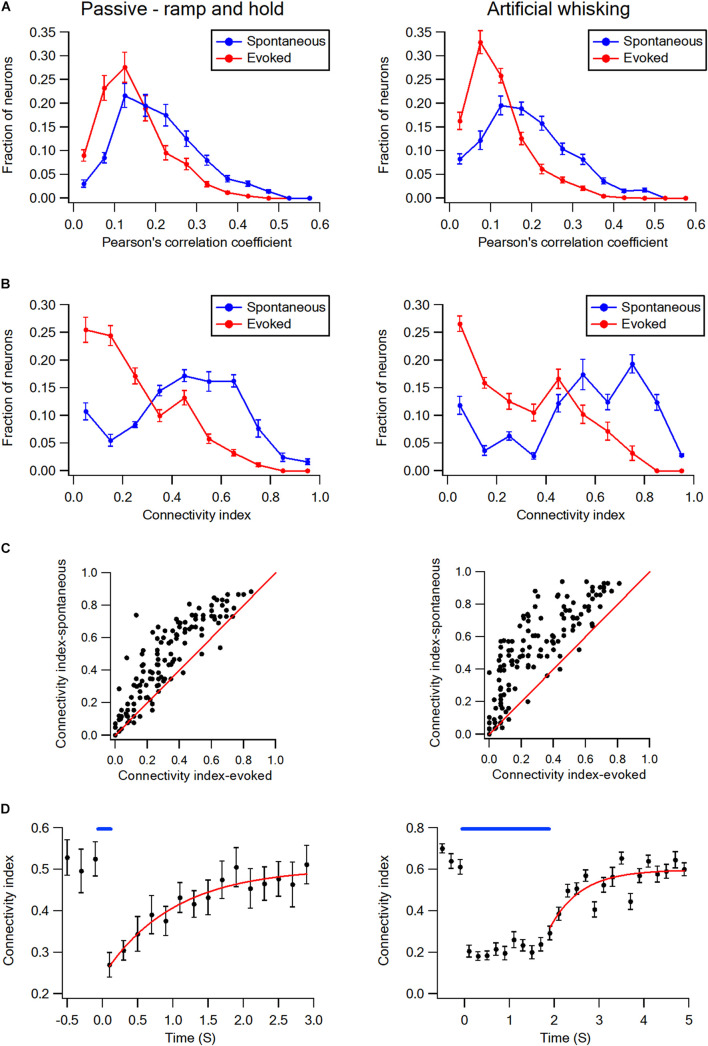
Desynchronization histograms and dynamics in wS1 barrel cortex. **(A)** Histograms showing the averaged fraction of wS1 neurons (*M* ± *SEM*) with different Pearson’s correlation coefficient values during ramp-and-hold (left) and artificial whisking (right) paradigms. Note the leftward shift of the curves during evoked activities. Comparison of the spontaneous and evoked histograms during both the passive ramp-and-hold and artificial whisking yielded *p* < 0.001 using the X^2^ statistical test. **(B)** Histograms showing the averaged fraction of wS1 neurons (*M* ± *SEM*) with different connectivity index during ramp-and-hold (left) and artificial whisking (right) paradigms. Note the leftward shift of the curves during evoked activities. Similarly, a leftward shift during the evoked state is observed (*n* = 213 units 7 rats and 209 units from 7 rats in passive deflection and artificial active whisking, respectively). Comparison of the spontaneous and evoked histograms during both the passive ramp-and-hold and artificial whisking yielded *p* < 0.001 using the X^2^ statistical test. **(C)** The connectivity indexes during spontaneous pre-stimulus and evoked states are shown for the individually recorded neurons. The diagonal line shows neurons in which the connectivity index was unaffected by stimulation. Note the connectivity index of the majority of neurons decreased with whisker stimulation during both the passive ramp-and-hold and artificial whisking paradigms. **(D)** Overall curves (regular and fitted) representing the averaged connectivity index (*M* ± *SEM*) dynamics in wS1 neurons before and after applying the passive whisker ramp-and-hold (left, 169 units from 5 rats) and artificial whisking (right, 173 units from 5 rats) stimulations. A mono-exponential function was fit to the graphs with time constants of 1.15 ± 0.33 s for ramp-and-hold stimulation and 1.27 ± 0.3 s for artificial whisking.

This is shown for both a subset of layer-2/3 neurons in a single experiment (Figures 1E,F) and the averaged results of all recorded neurons in all rats (Figures 1G,H). On the average, whisker activation resulted in a reduction of 30–40% in both the Pearson’s correlation coefficient and connectivity index as compared to the spontaneous pre-stimulus state (Figures 1G,H). The comparison of the Pearson correlation and connectivity index histograms between the spontaneous pre-stimulus and evoked states has revealed that both the whisker stimulation paradigms had resulted in a leftward shift of the histograms (Figures 2A,B). Comparing the stimulus and evoked connectivity index values of individual neurons revealed that whisker activation decreased the connectivity index of almost all individual neurons (Figure 2C). Interestingly, 98 ± 0.8% of connected neuronal pairs during the activated state were also functionally connected during the spontaneous pre-stimulus state. Thus, aside from infrequent instances, whisker activation resulted in the disconnection of neuronal pairs that were functionally connected during the spontaneous pre-stimulus state.

Upon the termination of the whisker activation with either the ramp-and-hold stimulation or artificial whisking, the connectivity index has gradually recovered back to the pre-stimulus control value (Figure 2D). Recovery of the connectivity index followed a mono-exponential function with an average time constant of approximately 1.2 s (1.15 ± 0.33 s following the ramp-and-hold stimulation, 169 units from 5 rats; 1.27 ± 0.3 s following the artificial whisking against sandpaper, 173 units from 5 rats).

**FIGURE 3:**
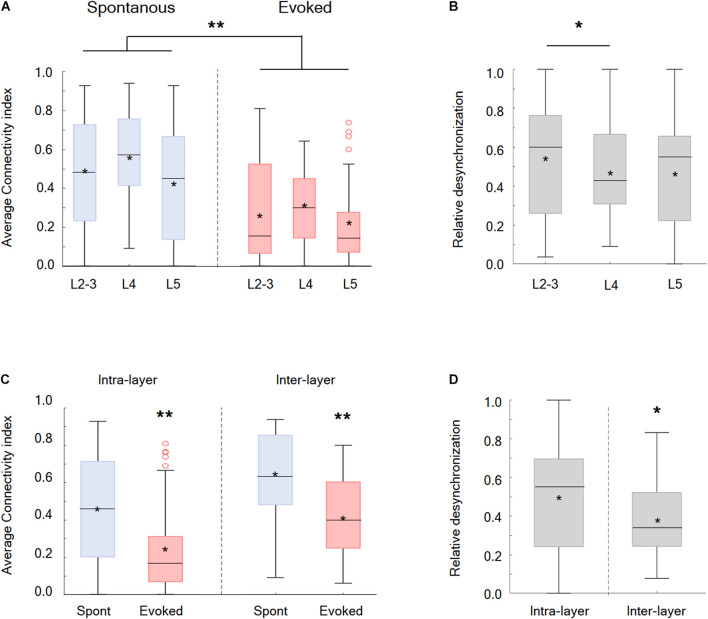
Desynchronization in the different cortical layers in the wS1 cortex and within different zones inside the layers. **(A)** Box plots of the connectivity index in spontaneous (blue) versus evoked (red) whisker activation in putative layers 2-3, 4, and 5 of the wS1 barrel cortex (209 neurons from 7 rats). The middle line in the box plots represents the median value and the asterixis represents the mean value. A value of **p < 0.01 is for the comparison of the data for each layer between spontaneous and evoked conditions. The comparison was obtained with the *t*-test or the Mann-Whitney non-parametric test when the data did not adhere to a normal distribution (L2-3 and L5 of the evoked conditions). Comparison between the layer under both the spontaneous and evoked states yielded no significant differences, using either the ANOVA or the Kruskal Wallis non-parametric test. **(B)** Box plots of the relative desynchronization [(Evoked-spont)/spont)] are presented for each putative cortical layer. The middle line in the box plots represents the median value and the asterixis represents the mean value. A value of *p < 0.05 was obtained with the Mann–Whitney non-parametric test. **(C)** Box plots of the connectivity index in spontaneous (blue) versus evoked (red) activities for neuronal pairs located within the same layer (intra-layer) and in different layers (inter-layer). The middle line in the box plots represents the median value and the asterixis represents the mean value. A value of *p < 0.05 obtained with either the Mann-Whitney non-parametric test (intra-layer) or the *t*-test (inter-layer). **(D)** Box plots of the relative desynchronization [(Evoked-spont)/spont)] is shown for functionally connected neurons located within the same layer (intra-layer) and in different layers (inter-layer). The middle line in the boxplots represents the median value and the asterixis represents the mean value. A value of *p < 0.05 was obtained with the *t*-test.

Afterward, the synchrony and functional connectivity were examined within and between different putative cortical layers. Our findings showed that the desynchronization, evoked by the whisker activation with either the passive ramp-and-hold or artificial whisking, was a global phenomenon involving all recorded wS1 neocortical layers and was observed for both neurons located within the same layer or in different putative layers (Figures 3C,D). However, two significant differences were observed between the layers. During the whisker activation, neuronal pairs within the putative layer 2/3 were more likely to functionally disconnect than the pairs located in layer 4 (Figures 3A,B). Finally, it is most interesting that the neuronal pairs located in different putative layers were more likely to functionally disconnect during whisker activation than the neurons located within the same layer (Figures 3C,D).

### Synchronization and Functional Connectivity of the wS2 Cortex During the Spontaneous and Evoked States

**FIGURE 4:**
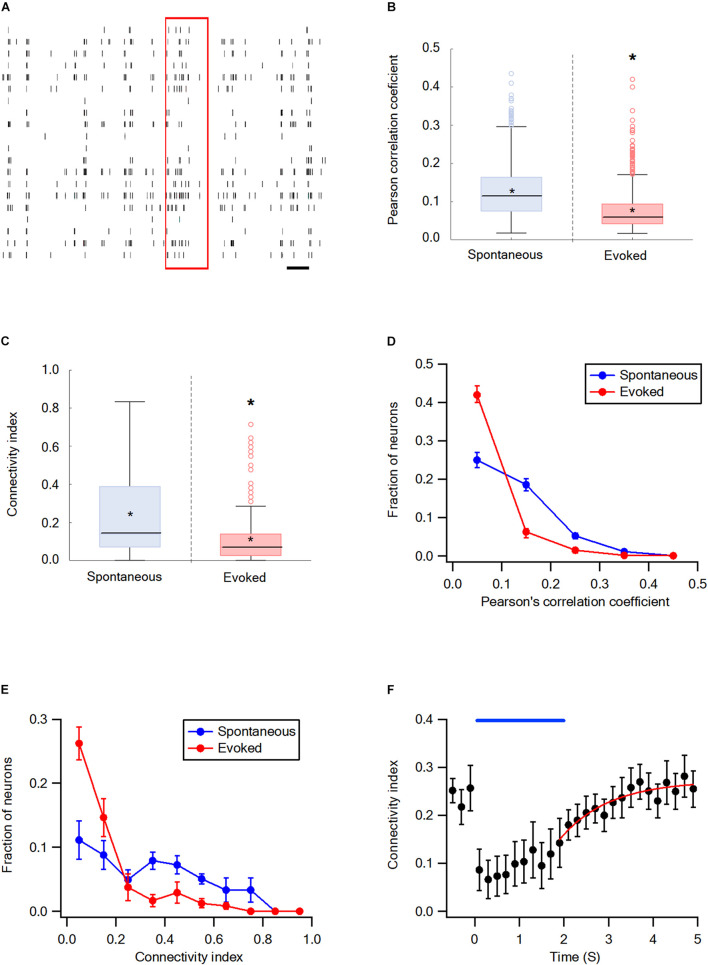
Desynchronization during evoked activity in the wS2 cortex. **(A)** Raster plots an example of spike trains during the single-trial from layer 2-3 neurons in the S2 cortex. The red rectangle represents the stimulus and the black scale bar represents 1 second. **(B)** Box plots of the Pearson’s correlation coefficient during spontaneous and evoked activity states (207 units from 6 rats). The middle line in the boxplots represents the median value and the asterixis represents the mean value. A value of *p < 0.05 was obtained with the *t*-test. **(C)** Box plots of the average connectivity index during spontaneous and evoked activity states. The middle line in the box plots represents the median value and the asterixis represents the mean value. A value of *p < 0.05 was obtained with the Mann-Whitney non-parametric test. **(D)** Histograms showing the average fraction of wS2 neurons (*M* ± *SEM*) with different Pearson’s correlation coefficient values during spontaneous and stimulus-evoked activity states. Comparison of the spontaneous and evoked histograms during both passive ramp-and-hold and artificial whisking yielded *p* < 0.001 using the X^2^ statistical test. **(E)** Histograms showing the averaged fractions of neurons (*M* ± *SEM*) with different connectivity index values during spontaneous and stimulus-evoked activity states. Comparison of the spontaneous and evoked histograms during both passive ramp-and-hold and artificial whisking yielded *p* < 0.001 using the X^2^ statistical test. **(F)** The averaged connectivity index (*M* ± *SEM*) in wS2 as a function of time before, during, and after artificial whisking mediated whisker activation (172 units from 5 rats). A mono-exponential function was fit to the graph with a time constant of 1.29 ± 0.3 s.

The synchrony and network connectivity in the wS2 cortex were examined next. For the wS2 experiments, we stimulated whiskers by artificially whisking the entire B whisker row against sandpaper since the ramp-and-hold and artificial whisking of a single whisker yielded very small responses. In general, the results in wS2 were like those in the wS1 cortex. Whisker stimulation markedly decreased the average Pearson’s correlation coefficient and connectivity index of wS2 neurons compared with the spontaneous pre-stimulus state (Figures 4A–C). The stimulation shifted the connectivity histograms to the left (Figures 4D,E) and 98.3 ± 0.1% of functionally connected wS2 neurons during the activated state were also functionally connected during the spontaneous pre-stimulus state. At the end of the artificial whisking, pairwise connectivity in wS2 gradually recovered following a mono-exponential time course with an average time constant of 1.29 ± 0.3 s (Figure 4F).

### Non-random Connectivity in the wS1 Cortex During the Spontaneous Pre-stimulus and Evoked States

**FIGURE 5:**
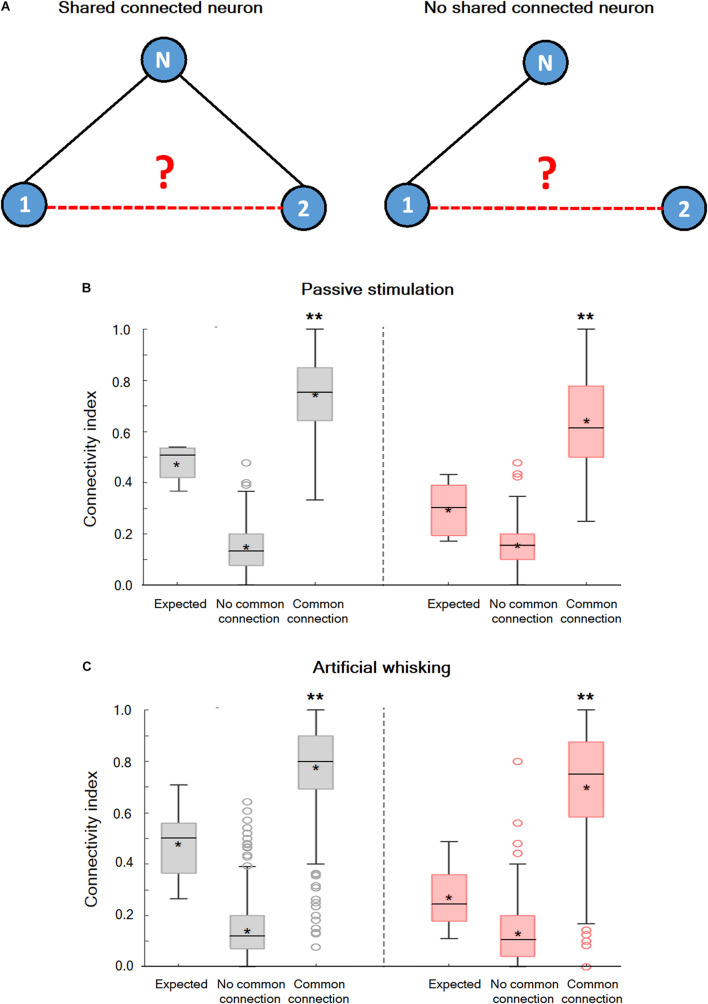
Non-random connectivity in the wS1 barrel cortex during the spontaneous pre-stimulus and activated states. **(A)** A Diagram of three hypothetical neurons explaining the concept of the performed analysis. In the left panel, units 1 and 2 share a common connection with unit N while in the right panel no common connection exists. **(B)** Boxplots of the connectivity index during spontaneous pre-stimulus (gray) and passive ramp-and-hold whisker activation (red) for neuronal pairs that share a common recorded connected neuron (common connection), neuronal pairs that do not share any common recorded connected neuron (no common connection), and the expected connectivity index assuming random connectivity (expected: 169 units from 5 rats). The middle line in the boxplots represents the median value and the asterixis represents the mean value. A value of **p < 0.01 was obtained with the *t*-test for comparison of neurons with common connections and neurons with no common connections, as well as ANOVA comparing all three groups. **(C)** Box plots of the connectivity index during spontaneous pre-stimulus (gray) and artificial whisking against sandpaper (red) for neuronal pairs that share a common recorded connected neuron (common connection), neuronal pairs that do not share any common recorded connected neuron (no common connection), and the expected connectivity index assuming random connectivity (173 units from 5 rats). The middle line in the boxplots represents the median value and the asterixis represents the mean value. A value of **p < 0.01 was obtained with the *t*-test for comparison of neurons with common connections and neurons with no common connections. *p < 0.05.

An examination of the connection probability between neurons that shared a third functionally connected neuron was carried out to investigate whether neurons in wS1 are randomly connected or alternatively form interconnected groups of neurons. In case subgroups of inter-connected neurons exist, we expected the connection probability between neurons that shared a third functionally connected neuron to be increased. Figure 5 shows that this is indeed the case. During both the spontaneous pre-stimulus and evoked states, the averaged connection probability between neuronal pairs that shared at least one connected neuron was significantly larger than the connection probability between neuronal pairs that did not share any commonly connected neuron, and the expected probability for random connectivity. This was true during the spontaneous pre-stimulus state, and even more so during the evoked state (Figures 5B,C). Therefore, during both the spontaneous pre-stimulus and evoked states, the wS1 cortex contained groups of interconnected neurons with increased intra-group connection probability.

### Inter-Neuronal Spatial Distance and Functional Connectivity in wS1 and wS2 During the Spontaneous Pre-stimulus and Evoked States

**FIGURE 6:**
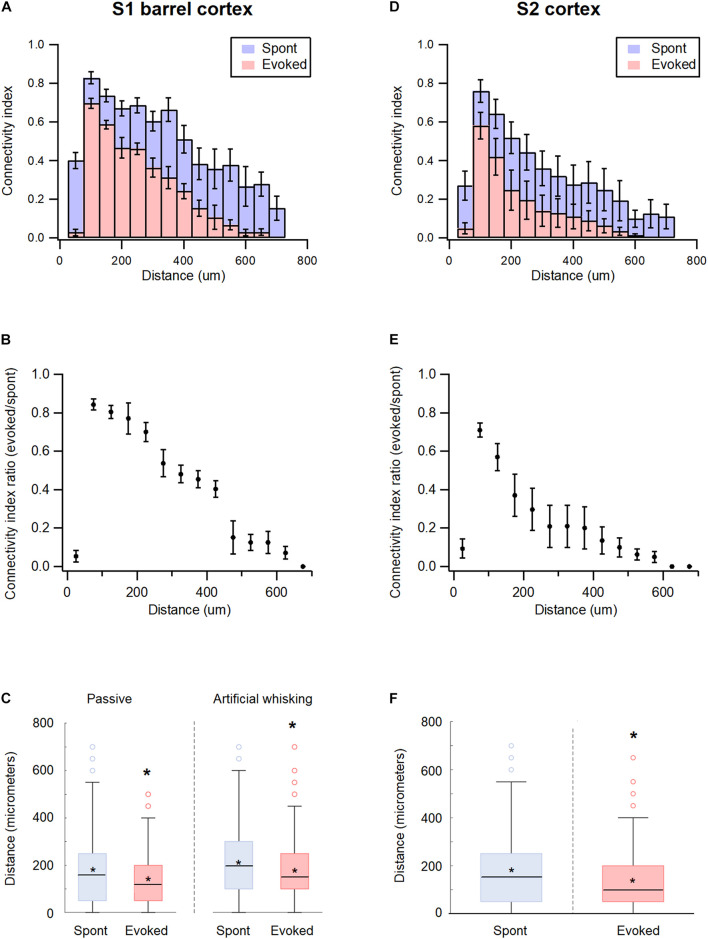
Distance dependency of functional connectivity in somatosensory cortices according to distance. **(A,D)** Histograms of the average connectivity index (*M* ± *SEM*) at different distance values between the recorded neuron pairs during spontaneous (blue) and evoked (red) activity states in wS1 **(A)** and wS2 **(D)** cortices. **(B,E)** Average (*M* ± *SEM*) ratio of connectivity index between the evoked and spontaneous activity states in wS1 **(B)** and wS2 **(E)** cortices. **(C)** Box plots of the distance between connected units in wS1 during the spontaneous (blue) and evoked (red) activity states for ramp-and-hold (passive) and artificial whisking against sandpaper (artificial whisking) whisker activation paradigms. The middle line in the boxplots represents the median value and the asterixis represents the mean value. A value of *p* < 0.05 was obtained with the *t*-test. **(F)** Box plots of the distance between connected units in wS2 during the spontaneous (blue) and evoked artificial whisking (red) mediated activity states. The middle line in the boxplots represents the median value and the asterixis represents the mean value. A value of *p < 0.05 was obtained with the *t*-test.

The study further investigated the effect of spatial distance between the recorded neurons on the pairwise functional connectivity during the spontaneous and activated states in the wS1 cortex. The probability for the pairwise functional connectivity during the spontaneous pre-stimulus state decreased with increasing distance between neurons, in inter-neuronal distances greater than 50 μm (Figure 6A). Interestingly, closely spaced neurons recorded with the same electrode demonstrated a relatively low probability of functional connections. This surprising finding may have resulted due to the condition that the spatial proximity of neurons was determined at the somatic level, while synaptic connections between neurons were determined at the axonal-dendritic level.

The whisker activation reduced functional connectivity between the neuronal pairs at all distances. However, longer connections were more likely to “disconnect” during the activated state (Figures 6A,B). For example, the passive ramp-and-hold whisker activation has reduced the average pairwise connectivity index of neurons located 50 μm and 200 μm apart by 13.5 ± 3.5 and 26.6 ± 6.1%, respectively (*p* < 0.01, 173 units from 5 rats). In addition, the averaged distance between functionally connected neurons decreased by 17.5 ± 3.1% when comparing the spontaneous pre-stimulus period with the ramp-and-hold whisker stimulation (Figure 6C).

Similar results were obtained in the wS2 cortex. During the spontaneous pre-stimulus state, the probability for functional connections between neuronal pairs in wS2 decreased with a distance beyond 50 μm (Figure 6D). Similar to wS1, artificial whisking of the B row against sandpaper reduced the pairwise connectivity of wS2 neurons at all distances, yet the longer connections were more likely to disconnect during stimulation (Figure 6E). Artificial whisking decreased the average pairwise connectivity index of wS2 neuronal pairs located 50 and 200 μm apart by 29.1 ± 3.7 and 70.3 ± 11.8%, respectively (*p* < 0.01, 171 units from 5 rats), and reduced the averaged distance between functionally connected neurons by 35.2 ± 5.1% (*p* < 0.05, 171 units from 5 rats) (Figure 6F).

### The Effect of Angular and Coarseness Tuning on Functional Connectivity in the Barrel wS1 Cortex During the Spontaneous and Evoked States

The study further investigated whether the tuning properties of neurons affected their probability to be functionally connected. In obtaining the results of such a hypothesis, we examined two different tuning properties of neurons, angular and coarseness tuning.

**FIGURE 7:**
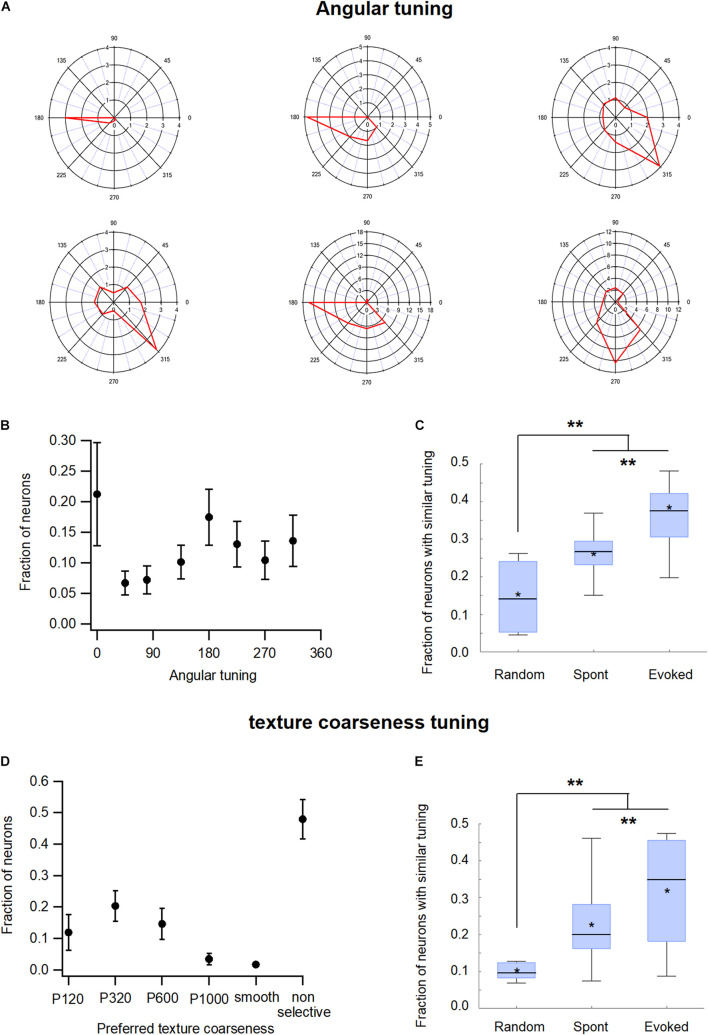
Functional organization of the barrel wS1 cortex: Functional tuning properties. **(A)** Polar plots of six individual wS1 neurons during ramp-and-hold vibrissa deflection to eight different angles (0°, 45°, 90°, 135°, 180°, 225°, 270°, and 315°). The average response of the neuron for each direction is plotted in red. **(B)** Distribution of the preferred angle in our neuronal population (213 units from 7 rats) presented as the average fraction of neurons (*M* ± *SEM*) preferring each of the eight angles in the ramp-and-hold passive stimulation. **(C)** Box plots of the fraction of functionally connected neurons, which share a similar preferred angle tuning measured during the spontaneous pre-stimulus and evoked activity states. In addition, the expected random value was calculated based on the distribution of preferred angles in each experiment. The middle line in the boxplots represents the median value and the asterixis represents the mean value. A value of **p < 0.01 was obtained with the *t*-test for comparison of each two groups, and the ANOVA for comparison of the three groups. **(D)** Distribution of the preferred coarseness in our recorded neuronal population (209 units from 7 rats) presented as the average fraction of neurons (*M* ± *SEM*) preferring each of the five coarseness tested (sandpaper with P120, P320, P600, and P1000 grades and a CD which was used as a smooth surface). **(E)** Box plots of the fraction of neurons sharing a similar preferred coarseness measured during spontaneous and evoked activities states. In addition, the expected random value was calculated based on the distribution of preferred coarseness in each experiment. The middle line in the boxplots represents the median value and the asterixis represents the mean value. A value of **p < 0.01 was obtained with the *t*-test or Mann-Whitney non-parametric test for comparison of each two groups, and the Kruskal Wallis non-parametric test for comparison of the three groups. *p < 0.05.

The neurons in the wS1 barrel cortex were tuned to the deflection angle of the whisker, a property termed angular tuning (Bruno et al., 2003; Kremer et al., 2011; Lavzin et al., 2012; Khateb et al., 2017). To investigate the relationship between angular tuning and functional connectivity in wS1, we activated the principal whisker (usually B2) through passive-piezo deflection in eight different directions. Figure 7A presents examples of the angular tuning curves of six different wS1 neurons, while Figure 7B presents the distribution of the preferred deflection angle across our recorded wS1 neuronal population. When we examined the functional connectivity between our neurons, we found that the neurons which shared the same preferred deflection angle were significantly more likely to be functionally connected. During the spontaneous pre-stimulus state the average probability of functional connections between neuronal pairs that shared the same preferred angle was 28.7 ± 6.6% compared with only 19.7 ± 6.1% expected probability from a random connection pattern (*p* < 0.01, Figure 7C; 213 units from 7 rats). Whisker stimulation further increased the probability of functional connections between neuronal pairs that shared the same preferred angle to 36.8 ± 6.4% (Figure 7C, *p* < 0.05 compared to the spontaneous pre-stimulus state).

Previously we have shown that the neurons in the wS1 barrel cortex were also tuned to surface coarseness (Garion et al., 2014). To investigate the effect of coarseness tuning on pairwise functional connectivity in wS1 neurons, we performed artificial whisking of the B2 principal whisker against sandpapers using five different degrees of coarseness (P120, P320, P600, P1000, and smooth surface). Figure 7D presents a histogram of the preferred coarseness in our recorded neuronal population. Similar to angular tuning, neurons that shared the same preferred coarseness tended to be functionally connected. During the spontaneous pre-stimulus state the average probability for functional connectivity between neuronal pairs that shared the same preferred texture was 22.5 ± 4.8% compared with 12.5 ± 2% expected probability from a random connection pattern (Figure 7E). Artificial whisking against sandpaper further increased the connection probability of neurons that shared the same preferred coarseness to 31.3 ± 5% (Figure 7E).

## Discussion

In this study, we investigated the effect of whisker activation on synchronization and functional connectivity of the primary wS1 and secondary wS2 somatosensory whisker cortices. This study has three main findings. First, during the spontaneous pre-stimulus state, neurons in the wS1 cortex were functionally connected in a non-random manner wherein connections between nearby neurons which shared similar tuning properties were favored. Second, the whisker activation with either the passive ramp-and-hold whisker deflection or artificial whisking against sandpaper has desynchronized the network and functionally disconnected approximately a third of the wS1 neuronal pairs that were connected during the spontaneous pre-stimulus state. Third, the whisker activation preferentially disconnected neuronal pairs that were further apart and possessed non-congruent tuning properties. Therefore, the whisker activation has both decreased the size of the small-world network topology of the cortical network and increased the functional uniformity within functional subnetworks of the activated wS1 barrel cortex. This study also found that similar to the wS1 cortex, the artificial whisking of a whisker row against sandpaper has desynchronized and reduced the functional connectivity between wS2 neurons, with preferential preservation of functional connections between nearby neurons.

Importantly, all our recordings were performed by a single shaft electrode in the vertical direction only. Thus, our conclusions were limited to synchronization and functional connectivity within the same barrel-cortical column. Further studies are needed to examine whether similar phenomena also occur within different barrel-cortical columns.

In this study, we used pairwise correlations as the basis for functional connectivity. Although correlated firing has suggested that the two neurons belong to the same functional network, synchronized firing can result from common excitatory or even inhibitory inputs, including subcortical inputs such as thalamocortical afferents. Moreover, correlations can evidently be context and state-dependent. This means that under certain conditions, cells are driven by the same inputs and show “functional connectivity,” while the same neurons in other conditions may be driven by different inputs and are “functionally disconnected.”

Synchronizations at different timescales are common phenomena in cortical networks. They were demonstrated both at the single cell level, using spike timing correlated firing of neuronal pairs, as well as at the field potential level, in which correlative oscillations at different frequency bands on electroencephalogram (EEG) and electrocorticogram (ECoG) recordings were reported (Roy and Alloway, 1999; Steinmetz et al., 2000; Bair et al., 2001; Fries et al., 2001; Kohn and Smith, 2005; Dong et al., 2008; Fries, 2009; Jermakowicz et al., 2009; Renart et al., 2010; Bruno, 2011; Hansen and Dragoi, 2011; Okun et al., 2015). Previous studies have shown that the synchronization within cortical networks has participated in various behavioral phenomena such as attention, perception, learning, and encoding of various features of visual stimuli (Steinmetz et al., 2000; Hansen and Dragoi, 2011; Hipp et al., 2011; Bosman et al., 2014).

Similar to the previous reports in rodents and primates, we also showed synchronized firing of neuronal pairs in the wS1 and wS2 cortices during both the resting and evoked states. Moreover, similar to our findings, previous studies in rodents and primates also showed that the pairwise synchronization was distance-dependent (Steinmetz et al., 2000; Renart et al., 2010; Okun et al., 2015) and was greater for the neuronal pairs which shared comparable tuning in both the V1 (Samonds et al., 2003; Kohn and Smith, 2005; Jermakowicz et al., 2009) and wS1 cortices (Khatri et al., 2009). Interestingly, in contrast to our findings, pairwise synchronization was not observed in putative layer 4 of V1 of monkeys (Smith et al., 2013).

One of the major findings of our study was a reduction in pairwise synchronization during whisker stimulation. This finding differed from the previous reports which described that the sensory stimulation has increased the pairwise synchronization in the barrel (Roy and Alloway, 1999) and primary visual cortices (Jermakowicz et al., 2009). The discrepancy between our findings and those reported by the study of Jermakowicz et al. (2009) may be related to the differences between the visual and somatosensory systems. The differences between our findings and those reported by the study of Roy and Alloway (1999) were harder to reconcile and were probably related to the fact we recorded along a vertical electrode from different depths of the same barrel, while the study of Roy and Alloway recorded from multiple single electrodes resulting in horizontally separated neurons. Differences in activation paradigms (air puffs vs. passive ramp-and-hold whisker activation and artificial whisking against sandpaper) may have also contributed to the differences between the studies.

The previous studies which examined the topology of the cortical network had reported neocortical networks, including that of the wS1 cortex in rodents exhibit rich-club small-world topology (Hilgetag and Kaiser, 2004; Bassett and Sporns, 2017; Schröter et al., 2017). Consistent with these reports, we also showed a small-world network topology in wS1. In addition, we showed that the whisker stimulation has further decreased the size of the small network topology and increased the functional uniformity of the network. In our study, we did not examine the existence of hub neurons or a rich-club topology in the somatosensory cortices.

In a set of seminal studies, the Petersen group described two cortical states in the wS1 network, quiet wakefulness, and active whisking states. During quiet wakefulness, layer 2/3 of excitatory pyramidal neurons and parvalbumin (PV) expressing interneurons have shown synchronized fluctuations of the membrane potentials. This was also manifested in the extracellular field potentials and neuronal firing. Interestingly, somatostatin (SST) expressing interneurons did not participate in this synchronized activity. In contrast, when the animal is actively whisking or performing a behavioral task, the wS1 network entered an active state where the membrane potential of different layer-2/3 pyramidal neurons and PV interneurons desynchronized (Crochet and Petersen, 2006; Poulet and Petersen, 2008; Gentet et al., 2012; Sachidhanandam et al., 2013; Eggermann et al., 2014; Petersen, 2019; Poulet and Crochet, 2019). The mechanisms underlying the transition between the two states probably involve both the thalamic and cholinergic inputs to the wS1 cortex (Poulet et al., 2012; Eggermann et al., 2014; Petersen, 2019; Poulet and Crochet, 2019; Gasselin et al., 2021). We hypothesized that the reduced synchronized firing which we observed during whisker activation is related to the state transition that was previously reported by the Petersen group. Interestingly, we showed that this phenomenon was not restricted to layer 2/3 of the wS1 cortex, but also occurred in layers 4 and 5 of wS1 and wS2 as well (see also Zhao et al., 2016).

Theoretical studies that used information theory to investigate synchronization raised the concern that correlated firing of neurons may be detrimental for population coding of information by decreasing information content of the network (Zohary et al., 1994; Abbott and Dayan, 1999; Barlow, 2001; Simoncelli and Olshausen, 2001; Kohn and Smith, 2005). More recent work partially addressed this enigma and showed that only differential correlations could cause information saturation; balanced excitatory-inhibitory activation could assist in preserving information content even with near-zero correlative firing (Salinas and Sejnowski, 2001; Renart et al., 2010; Kohn et al., 2016). Despite these revelations, correlative firing yet still limits to some extent the information capacity of neuronal populations (Moreno-Bote et al., 2014; Bartolo et al., 2020). In our study, we proposed an additional route for increasing the sensory information content of the cortical network. We showed that during the whisker stimulation, when afferent sensory input information is received and processed by the cortex, the cortical network undergoes partial desynchronization. In turn, this partial desynchronization of the wS1 and wS2 networks increases their capability to compute and encode the incoming somatosensory information. Interestingly, previous studies reported that the correlative firing is also reduced by increased attention and arousal (Steinmetz et al., 2000; Poulet and Petersen, 2008; Cohen and Maunsell, 2009; Mitchell et al., 2009; Herrero et al., 2013; Poulet and Crochet, 2019), as well as by adaptation to the whisker stimulation (Khatri et al., 2009). It is possible that similar to the whisker activation, network desynchronization under these conditions could also be used to increase information content and computational capacity of the cortical network.

One of the major limitations of our study was the use of anesthetized rats since anesthesia could increase the synchrony within the cortical network. Working under anesthesia allowed us to investigate well-controlled and repeated robust activation of whiskers. Nevertheless, both the anesthesia and the circumstance that the rats were not actively performing the task may have influenced the activity and synchronization (Constantinople and Bruno, 2011; Lissek et al., 2016; Lee et al., 2020). Future experiments are required to corroborate our findings in awake behaving rodents.

## Data Availability Statement

Most original contributions presented in the study are included in the article/Supplementary Material. Additional raw data supporting the conclusions of this article will be made available by the authors, without undue reservation.

## Ethics Statement

The animal study was reviewed and approved by Technion, Haifa Israel.

## Author Contributions

MK conducted all experiments and analysis, assisted in conceiving the project, planning the experiments, and writing the manuscript. JS assisted in conceiving the project, planning the experiments, supervising the project, and writing the manuscript. YS conceived the project, planned most the experiments, supervised the project, and wrote most of the manuscript. All authors contributed to the article and approved the submitted version.

## Conflict of Interest

The authors declare that the research was conducted in the absence of any commercial or financial relationships that could be construed as a potential conflict of interest.

## Publisher’s Note

All claims expressed in this article are solely those of the authors and do not necessarily represent those of their affiliated organizations, or those of the publisher, the editors and the reviewers. Any product that may be evaluated in this article, or claim that may be made by its manufacturer, is not guaranteed or endorsed by the publisher.

## Footnotes

1
www.graphpad.com
