# Supplementary material for: State-Dependent Synchrony and Functional Connectivity in the Primary and Secondary Whisker Somatosensory Cortices
Source: Front Syst Neurosci. 2021 Sep 20;15:713397. doi: 10.3389/fnsys.2021.713397 (PMC8489558; doi:10.3389/fnsys.2021.713397)
Supplement: Supplementary file 1 [file Data_Sheet_1.pdf]

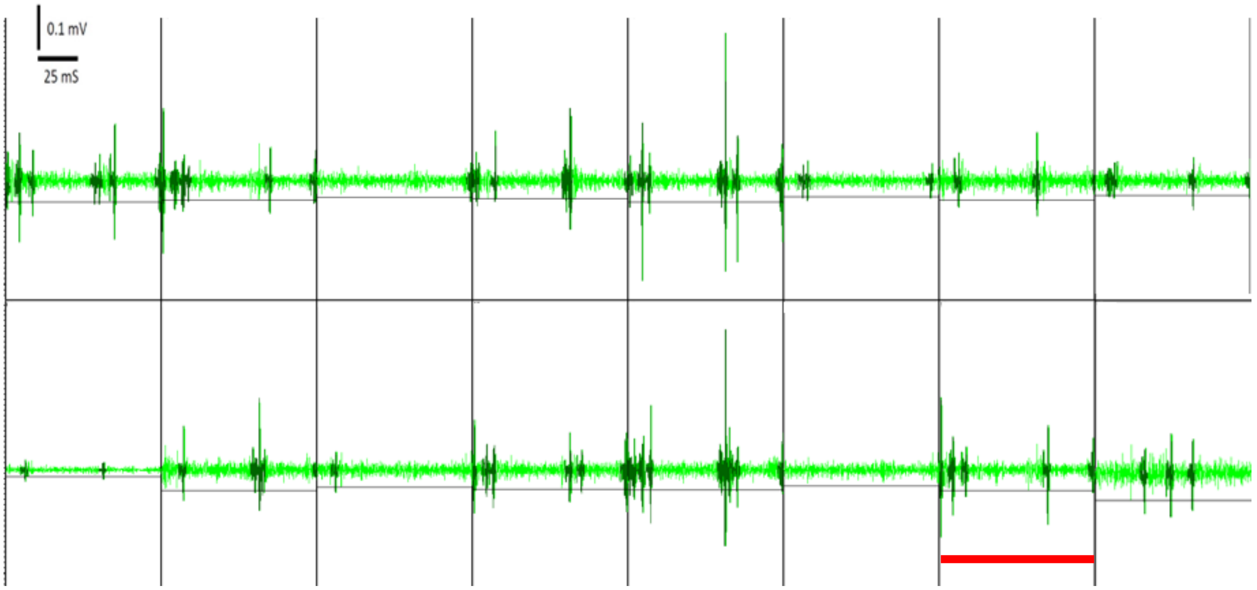

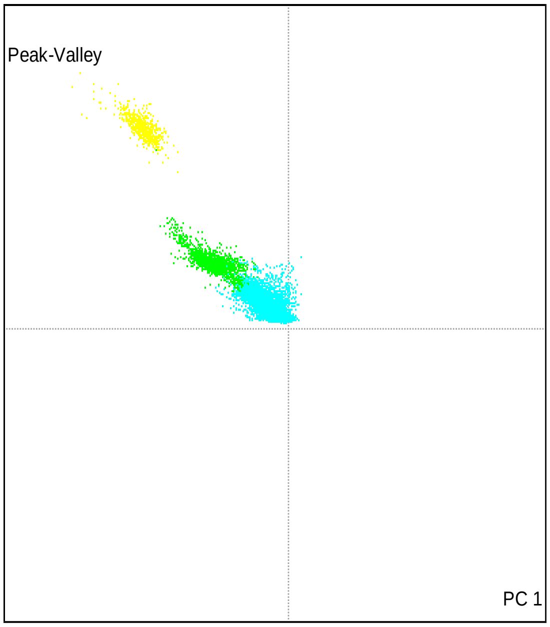

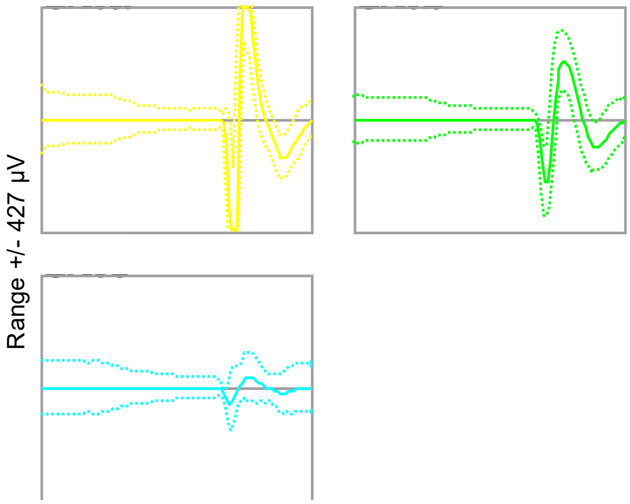

Multivariate ANOVA

**In 2D Cluster Space:**	**F(4,39468) =**	66.003534	9.59309e-056
**In 3D Cluster Space:**	**F(6,39466) =**	-1.#IND00	-1

**Cluster Validity**	**2D**	**3D**
**J3 :**	6.63173	6.03452
**Pseudo-F :**	284113	258528
**Davies-Bouldin :**	0.145026	0.152181
**Dunn :**	3.09651	3.22196

Supplementary Figure 1: Spike sorting and cluster validity. The figure demonstrates our spike sorting and cluster validity method. The data is obtained from the offline sorting software used for our analysis (OFS by Plexon). The upper panel shows a raw trace with 16 recording channels, and the red line marks the channel in which sorting is demonstrated in the panels bellow. A threshold placed 3.5 SD bellow the baseline detected multiunit data (marked as black lines on the trace). Note this threshold probably misses some multi units with amplitudes smaller than 3.5 below the baseline. The left middle panel demonstrates the peak amplitude of individual spikes plotted as a function of the Eigenvalue of the first component obtained with primary component analysis (PC1). The individual spikes are subdivided to three different units (marked as yellow, green and blue). The three right panels show the average waveform of the three units sorted in the traces bellow. The numbers presented bellow represents the values of the multi-variant ANOVA and the four statistical analysis methods used to validate clustering of the presented data.
